# Estimate of Fukushima-derived radiocaesium in the North Pacific Ocean in summer 2012

**DOI:** 10.1007/s10967-018-6249-7

**Published:** 2018-11-10

**Authors:** Y. Inomata, M. Aoyama, T. Tsubono, D. Tsumune, Y. Kumamoto, H. Nagai, T. Yamagata, M. Kajino, Y. T. Tanaka, T. T. Sekiyama, E. Oka, M. Yamada

**Affiliations:** 1 Institute of Nature and Environmental Technology, Kanazawa University, Kanazawa, 920-1192 Japan; 2 Institute of Environmental Radioactivity, Fukushima University, 1 Kanayagawa, Fukushima, 960-1192 Japan; 3 Environmental Science Research Laboratory, Central Research Institute of Electronic Power Industry, 1646, Abiko, 270-1194 Japan; 4 Japan Agency for Marine-Earth Science and Technology, 2-15 Natsushima-cho, Yokosuka, 237-0061 Japan; 5 Nihon University, 40-25-3 Sakurajosui, Setagaya, Tokyo, 156-8550 Japan; 6 Meteorological Research Institute, 1-1 Nagamine, Tsukuba, 305-0052 Japan; 7 Atmosphere and Ocean Research Institute, The University of Tokyo, Kashiwa, 277-8564 Japan; 8 Institute of Radiation Emergency Medicine, Hirosaki University, Hirosaki, 036-8564 Japan

**Keywords:** Radiocaesium, FNPP1 accident, Central mode water, Subtropical mode water, Inventory, North Pacific Ocean, Mass balance

## Abstract

Distributions of radiocaesium (^134^Cs and ^137^Cs) derived from the Tokyo Electric Power Company (TEPCO) Fukushima Dai-ichi Nuclear Power Plant (FNPP1) accident in the North Pacific Ocean in the summer of 2012 were investigated. We have estimated the radiocaesium inventory in the surface layer using the optimal interpolation analysis and the subducted amount into the central mode water (CMW) by using vertical profiles of FNPP1-^134^Cs and mass balance analysis as the first approach. The inventory of the ^134^Cs in the surface layer in the North Pacific Ocean in August–December 2012 was estimated at 5.1 ± 0.9 PBq on 1 October 2012, which corresponds to 8.6 ± 1.5 PBq when it was decay corrected to the date of the FNPP1 accident, 11 March 2011. It was revealed that 56 ± 10% of the released ^134^Cs into the North Pacific Ocean, which was estimated at 15.3 ± 2.6 PBq, transported eastward in the surface layer in 2012. The amount of ^134^Cs subducted in the CMW was estimated to be 2.5 ± 0.9 PBq based on the mass balance among the three domains of the surface layer, subtropical mode water, and CMW.

**Electronic supplementary material:**

The online version of this article (10.1007/s10967-018-6249-7) contains supplementary material, which is available to authorized users.

## Introduction

As a result of an extraordinary earthquake and subsequent giant tsunami on 11 March 2011, Tokyo Electronic Power Company Fukushima Dai-ichi Nuclear Power Plant (hereafter referred to as FNPP1), locate 37.42°N, longitude 141.03°E, received serious damage. Because of the damaged FNPP1 reactors, large amounts of radiocaesium (^137^Cs, with a half-life of 30.2 years and ^134^Cs, with a half-life of 2.06 years) were directly released into the North Pacific Ocean by atmospheric deposition and direct discharge of liquid-contaminated stagnant water from the FNPP1 accident site, mostly in March and April 2011 [1, 2]. It was recorded that extremely high radiocaesium activity concentrations reaching 6.8 × 10^7^ Bq m^−3^ were observed on 6 April 2011 at the site of the FNPP1 accident [3, 4]. From April to May 2011, a basin-scale assessment indicated that the FNPP1-derived radiocaesium activity concentrations in the surface waters in the North Pacific Ocean ranged from a few to 1000 Bq m^−3^ [1, 5]. Although the radiocaesium released by atmospheric deposition and direct discharge of liquid-contaminated stagnant water into the North Pacific Ocean by the FNPP1 accident were estimated by numerous studies, the median values of the released amount tended to converge on a range of 15–20 PBq [6, 7]. The directly discharged radiocaesium in the liquid-contaminated stagnant water into the North Pacific Ocean was estimated to be 3.5 ± 0.7 PBq using the ^131^I/^137^Cs activity ratio, which is a useful tracer to distinguish the release pathways between direct release and atmospheric deposition [2, 3]. The total ^134^Cs inventories ranged from 15.2 to 18.3 PBq [6]. This estimation is in good consensus with the ^134^Cs inventories estimated by other methods, namely, 15.3 ± 2.6 PBq by optimal interpolation analysis [8] and 16.1 ± 1.4 PBq by the North Pacific model [9]. It is considered that more than 75% of the radiocaesium released into the atmosphere from the FNPP1 event was deposited into the North Pacific Ocean.

Before the FNPP1 accident, ^137^Cs already existed in the North Pacific Ocean due to the large-scale atmospheric nuclear weapons testing that occurred in the late 1950s and early 1960s and the Chernobyl accident in 1986 [10–13]. In the 2000s, ^137^Cs activity concentrations were 1.5–2 Bq m^−3^ with almost homogenous distribution; however, relatively high activity, which exceeded 2 Bq m^−3^, was observed in the western part of the subtropical gyre in the North Pacific Ocean [11]. Because of its shorter half-life, ^134^Cs derived from the global fallout by large-scale nuclear weapons tests had already decayed in the seawater by 1993 [14]. ^134^Cs, therefore, is regarded as an adequate chemical tracer released from the FNPP1 accident to investigate the transport of the FNPP1-derived contaminated seawater in the North Pacific Ocean. Taking into account that the ^134^Cs/^137^Cs activity ratio originating from the FNPP1 accident was almost 1 [15, 16], we can consider that FNPP1-^137^Cs caused an additional increase of approximately 22–27% in the ^137^Cs inventory that existed in the North Pacific prior to the FNPP1 accident, which was estimated to be 69 PBq from nuclear weapons testing [6].

The basin-scale assessment indicated that the directly discharged and atmospheric deposited FNPP1-^134^Cs in the North Pacific Ocean was transported eastward in the surface layer by the Kuroshio and Oyashio Extensions and the North Pacific Current. In the summer of 2012, approximately 1.5 years after the FNPP1 accident, the main body of radioactive contaminated seawater was observed in the region of 40°N–45°N between 165°E and 170°W in the central North Pacific Ocean [17]. The zonal speed of FNPP1 radiocaesium in the surface water at mid-latitude in the North Pacific Ocean was estimated to be approximately 8 cm s^−1^ until March 2012 [1, 5]. The FNPP1-^134^Cs was further transported eastward and to a higher latitude as subarctic currents with a zonal speed of 3.5 cm s^−1^ from March 2012 to August 2014 [6]. The FNPP1-^134^Cs approached North America and diverged into the northward flowing Alaska Current and the southward flowing California Current beginning in February 2013 [18–20]. The arrival of FNPP1-radionuclides off the west coast of North America, on the Canadian continental shelf, was first reported in February 2015 [19, 20]. The North Pacific model also reproduced the eastward transport of FNPP1 radiocaesium in the North Pacific Ocean [9]. In addition, FNPP1 radiocaesium was found south of the Kuroshio Front, with a subsurface maximum at a depth of approximately 200–600 m in the western subtropical region [1, 18, 21–23]. The subsurface maxima of ^137^Cs existed in the subtropical mode water (STMW) with a potential water density anomaly (*σ*_θ_) = 25.0–25.6 kg m^−3^ [24] and the central mode water (CMW) with *σ*_θ_ = 26.0–26.5 kg m^−3^ [25, 26]. The STMW is formed as a deep winter mixed layer south of the Kuroshio and Kuroshio Extension, which is located north of approximately 28°N from ~ 132°E to the dateline [27–29]. The CMW is characterized by a pycnostad in the lower pycnocline in the North Pacific subtropical gyre. The CMW is formed in the deep water mixed layer located north of the Kuroshio Extension [30, 31]. The subsurface peak of FNPP1 radiocaesium in the STMW suggests that atmospheric deposition occurred in the formation region of STMW, then subduction into the ocean interior occurred during the southwestward transport within 10 months after the FNPP1 accident [17]. Kaeriyama et al. [23] estimated the amount of FNPP1-^134^Cs in the STMW at 4.2 ± 1.1 PBq in October–November 2012. However, the FNPP1 radiocaesium amount subducted into the CMW has not exactly been estimated.

The primary objectives of this study were as follows: (i) to investigate the distributions of radiocaesium in the surface seawater, (ii) to estimate the radiocaesium inventory in the surface layer, and (iii) to estimate the FNPP1 radiocaesium amount subducted into the CMW in the North Pacific Ocean in 2012. Furthermore, we discuss the fate of FNPP1 radiocaesium subducted into the ocean interior.

## Experimental

### Data

To elucidate the distributions of the radiocaesium activity concentrations, it is necessary to use as many data points as possible. We, therefore, compiled the data available from the literature and the monitoring results. Most of the data before the FNPP1 accident were included in the database, “Historical Artificial Radionuclides in the Pacific Ocean and its Marginal Seas (HAM database)” [32]. The data observed after the FNPP1 accident were also shown in Aoyama et al. [1]. Monitoring data were also available to use in this analysis [33–35].

In this study, we focused on the distribution of the central North Pacific Ocean in 2012, because the centre of the radioactive contaminated seawater in the surface layer was observed in the central region of the North Pacific Ocean, at approximately 180° in August–December 2012 [21]. The data used in this analysis consisted of 236 records for ^134^Cs and 343 records for ^137^Cs. These records are listed in the supplementary Table S1. The activity concentrations of radionuclides in seawater are radioactive decay corrected to 1 October 2012 and 11 March 2011. The term “surface seawater” used in this study is defined as a sample collected at less than a 10 m depth. The vertical profiles of radiocaesium activity concentrations were used to estimate the radiocaesium inventory. The seawater was collected at 5 stations from 5 to 1006 m in depth between August and September 2012 during the research cruise of “Hakuho-maru” (HK12-4). The detection limits of ^134^Cs and ^137^Cs in the seawater samples collected by HK12-4 were 0.17 and 0.28 Bq m^−3^, respectively. In addition, we used vertical profile data observed on 8 September 2012 during the research cruise of “Mirai” (MR12-E03) reported by Kumamoto et al. [17]. The detection limits of ^134^Cs and ^137^Cs were 0.1 and 0.17 Bq m^−3^, respectively. The details of the measurement were described in Kumamoto et al. [17, 18]. The activity concentrations of the radiocaesium in seawater are radioactive decay corrected to 11 March 2011. The seawater samples for obtaining the vertical profiles were collected at 58 points and are listed in the supplementary Table S2.

### Optimal interpolation analysis methods

Optimal interpolation analysis (OI) is an effective analytical scheme for depicting the horizontal distributions of irregularly distributed measurement data to gridded analytical value [36]. Detail of the analytical method has been already described in Inomata et al. [8, 13]. ^134^Cs and ^137^Cs activity concentrations estimated by OI (hereafter OICs134 and OICs137) were produced on a grid with a horizontal resolution of 3° longitude × 2° latitude, because the area is approximately equal to the geodetical area of one grid exactly, and the meridional transport prevails in the analytical area associated with the Kuroshio Current. The data used in the OI analysis were selected within the effective radius *R*; in other words, the data used in the OI analysis was located inside the circle with *R*. The background error at the observation point (*σ*_ο_) was considered. Based on a higher correlation coefficient for each gridded value estimated by the Akima spline method [37], which is also used as a conventional interpolation method and a gridded algorithm, and the OI, we selected 800 km and 100 as optimal values of *R* and *σ*_o_, respectively [8].

### Estimation of the water column inventory

The water column inventories of ^134^Cs were calculated as follows:

1 $$ {{\text{Total}}\;{\text{OICs}}134\;{\text{inventory}} = \sum\limits_{i = 1}^{n} {\left[ { {\text{OICs134}}\; \times \;{\text{sea}}\;{\text{area}}} \right]\;{\text{for}}\;{\text{each}}\;{\text{grid}}\;{\text{in}}\;{\text{the}}\;{\text{North}}\;{\text{Pacific}}\;{\text{Ocean}}\; \times \;{\text{mean}}\;{\text{penetration}}\;{\text{depth}}} } $$

where the OICs134 were the OI analytical values in each grid, the sea area was estimated based on the global relief model of Earth’s surface (ETOPO) (https://www.ngdc.noaa.gov/mgg/global/etopo1sources.html), and *n* was the grid number that was obtained from the OI analytical value. The mean penetration depth (MPD) of radiocaesium was used by the six vertical profiles of the ^134^Cs activity concentration data. The MPD, which is regarded as an indicator of the one-dimensional penetration of the tracer into the ocean [38], was estimated by the slopes of the relationship between water column inventories of ^134^Cs and ^134^Cs activity concentrations in the surface layer. The water column inventories of ^134^Cs were calculated by the simple trapezoidal integration of the activity concentration versus the depth profiles. Most ^134^Cs activity concentrations below a depth of 200 m were less than the detection limit. In the case of ^137^Cs, the ^137^Cs activity concentrations are gradually decreased with an increasing depth below 200 m; these concentrations originate from the sum of the FNPP1 accident and global fallout by large-scale atmospheric nuclear weapon tests. Considering vertical profile of ^134^Cs activity concentrations, ^137^Cs below a depth of 200 m would mainly originate from global fallout by large-scale atmospheric nuclear weapon tests. ^137^Cs data for the inventory estimation were used as the same depth data for the estimation of the ^134^Cs inventory. The relationship between the water column inventory and the ^134^Cs activity concentrations in the surface seawater indicated that the calculated slopes of these were 144 ± 15 m in the summer of 2012.

Based on the estimated MPD, the water column inventory was calculated based on the assumption that radiocaesium is distributed constantly from 0 to 72 m and decreases monotonically from a depth of 72–144 m. The error in the OICs134 and OICs137 inventory was estimated as the square root of the sum of the analytical standard deviation, statistical error of OI, and error of mixed penetration depth estimation.

### Mass balance analysis

To understand the environmental impact of released radiocaesium from FNPP1 into the North Pacific Ocean, the mass balance was considered as follows [39]:

2 $$\Sigma R_{i} =\Sigma I_{j}$$

where *R*_*i*_ is a released amount to each domain, and *I*_*j*_ is an inventory in each domain. Additionally, *i* is represented as follows: 1 = the deposition amount from the atmosphere into the ocean, and 2 = the direct discharged amount in the liquid-contaminated stagnant water, and *j* is represented as follows: 1 = the surface layer, 2 = STMW, 3 = CMW, 4 = sediment, and 5 = biota. Therefore, the mass balance was described as follows:

3 $$R_{1} + R_{2} = I_{1} + I_{2} + I_{3} + I_{4} + I_{5}$$

*R*_1_ was estimated to be 11.7–14.8 PBq [6], which was consistent with the global ocean model estimate of 10.5 ± 0.9 PBq by Tsubono et al. [9]. *R*_2_ was estimated to be 3.5 ± 0.7 PBq [4]. The inventory in the North Pacific Ocean, which is the sum of the atmospheric deposition and direct release amount as shown in *R*_1_ + *R*_2_, was estimated in several studies to be within a range of 15.2–18.3 PBq [6, 8, 9]. We used 15.3 ± 2.6 PBq as *R*_1_ + *R*_2_ based on the OI analysis by the authors [8]. The *I*_1_ was estimated by the OI analysis in this study. *I*_2_ was estimated to be 4.2 ± 1.1 PBq in previous study [23]. We can ignore the amount of *I*_4_, because the small amount of radiocaesium in the sediment was reported to be approximately 130 ± 60 TBq [40, 41]. We can also ignore *I*_5_ because the maximum estimate of radiocaesium in biota was 200 GBq based on the fish catch amount of 20 × 10^6^ kg around Fukushima and assuming an activity of 1 × 10^4^ Bq kg^−1^ [39]. Finally, we can simplify the Eq. (2) to indirectly estimate the amount of radiocaesium in the CMW based on the mass balance as shown below:

4 $$I_{3} = \left( {R_{1} + R_{2} } \right){-}I_{1} {-}I_{2}$$

### The atmospheric chemical transport model and physical parameters in the North Pacific Ocean

It is very difficult to investigate the radiocaesium deposition into the North Pacific Ocean. To estimate the deposition amount of radiocaesium into the mode water formation region in the North Pacific Ocean, we used the following three atmospheric chemical transport models taking into account the uncertainty among the different models: the Model of Aerosol Species IN the Global Atmosphere (MASINGAR I), its newer version (MASINGAR MK-II), and the Meteorological Research Institute—Passive-tracers Model for radionuclides (MRI-PM/r). The simulation settings are similar to those of Aoyama et al. [6]; therefore, we have refrained from repeating the details here.

MASINGAR I is a global aerosol transport model, which is coupled online with atmospheric general circulation models (AGCMs) of the Japan Meteorological Agency (JMA) [42]. MASINGAR I is coupled with an AGCM called MRI/JMA 98 [43] and has been used as the operational dust forecasting model by the JMA. The model resolutions were set to a T106 Gaussian horizontal grid (approximately 1.125° × 1.125°) and 30 vertical layers from the surface to a height of 0.4 hPa. MASINGAR MK-II is also coupled with the global atmospheric model [44] as a component of the Earth system model of the Meteorological Research Institute, the MRI-Earth System Model [45]. The model resolutions were set to a TL319 horizontal grid (approximately 0.5625° × 0.5625°) and 40 vertical layers from the ground surface to a height of 0.4 hPa. The MRI-PM/r is a regional-scale offline coupled meteorology-aerosol chemical transport model that was developed based on Kajino et al. [46]. The Advanced Research Weather Research and Forecasting (WRF) model [47] was used to simulate the meteorological data. The global analysis data provided by the National Centers for Environmental Prediction (NCEP), ds083.2 (1° × 1°, 6-HR time interval), were used for the initial and boundary conditions of the WRF and for the analysis nudging method. The horizontal grid resolution is 60 km with a Mercator map projection covering the North Pacific Ocean and the surrounding countries. And they are vertically 20 layers from the ground to 10 km in height with a terrain-following coordinate.

The *σ*_θ_ was investigated using the basin-scale model of the Japan Coastal Ocean Predictability Experiment (JCOPE2). JCOPE2 provided physical parameters such as potential temperature, salinity, zonal velocity, and meridional velocity of the oceanography. The data was generated from the western North Pacific Ocean (10.5–62°N, 108–180°E) with a horizontal resolution of 1/12°. The JCOPE2 data assimilates the remote-sensing data of altimetry and surface temperature and in situ data of temperature and salinity profiles [48]. The data from 21 March to 31 May, 2011 were used in this study. The *σ*_θ_ value corresponds to the STMW and CMW of the formation region, which were set to 25–25.6 and 25.8–26.4 kg m^−3^, respectively.

## Results

### Distribution and inventory of radiocaesium in the surface layer from August to December 2012

**Fig. 1:**
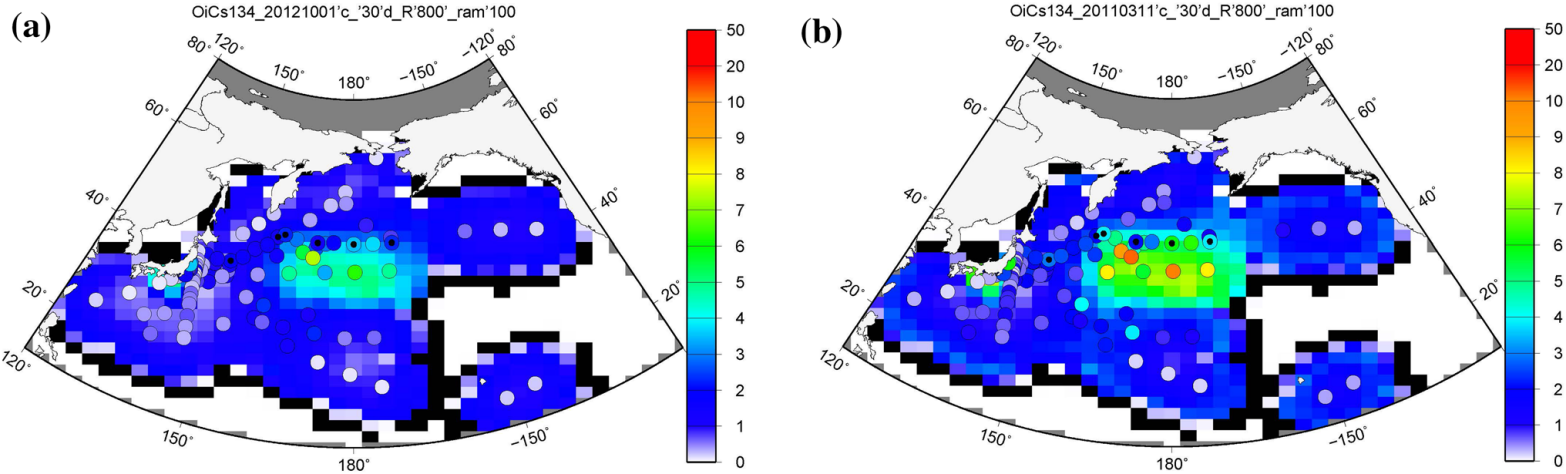
The distributions of ^134^Cs activity concentrations in the North Pacific Ocean in August–December, 2012. The data measured during the period from August to December, 2012 were composited and decay corrected to **a** 1 October 2012, **b** 11 March 2011. The circles are the measurement data. The circles with small black dots are sites measuring the vertical distribution. The unit of measurement is Bq m^−3^

Figure 1 shows the distributions of the ^134^Cs activity concentrations analysed using OI (OICs134) in the surface seawater from August to December 2012. The data measured in the period from August to December 2012 were compiled and decay corrected to 1 October 2012 (Fig. 1a), which is the middle day of the measurement, and 11 March 2011 at the time of the FNPP1 accident (Fig. 1b). The peak of OICs134 in the surface seawater was observed in the central North Pacific Ocean at approximately 40°N and 50°N between 165°E and 170°W. A similar distribution was also found in the OICs137 as shown in Fig. SI1a and 1b. In the region of high radiocaesium activity concentrations, the ^134^Cs/^137^Cs activity ratios were approximately 0.8–0.9. These ratios are almost the same value of the ratio in the radionuclide-contaminated stagnant water in the FNPP1, that is, the ratio was consistent from the core to the stagnant water [16]. A very close ^134^Cs/^137^Cs activity ratio to the source region in the central North Pacific Ocean suggests that the main body of the FNPP1 radiocaesium originated from the FNPP1 and was transported eastward along the surface current. The effect of dilution and subduction of ^134^Cs would be small during transport in the surface seawater. The estimated transport speed by the OICs134 activity concentrations that reached a longitude of 180° and 40°N, where the centre of the FNPP1-radiocaesum transport is, was approximately 8.5 cm s^−1^ by the OI analysis. This was consistent with the previous estimation of 8 cm s^−1^ by Aoyama et al. [5] and zonal speeds derived by Argo floats at the region. The estimated inventory of OICs134 is 5.1 ± 0.9 PBq with decay corrected to 1 October 2012 and 8.6 ± 1.5 PBq with decay corrected to 11 March 2011. For the inventory of OICs137, it was estimated to be 13.3 ± 2.3 PBq with decay corrected to 1 October 2012 and 13.9 ± 1.8 PBq with decay corrected to 11 March 2011.

### Radiocaesium deposition in the mode water formation region in the North Pacific Ocean

**Fig. 2:**
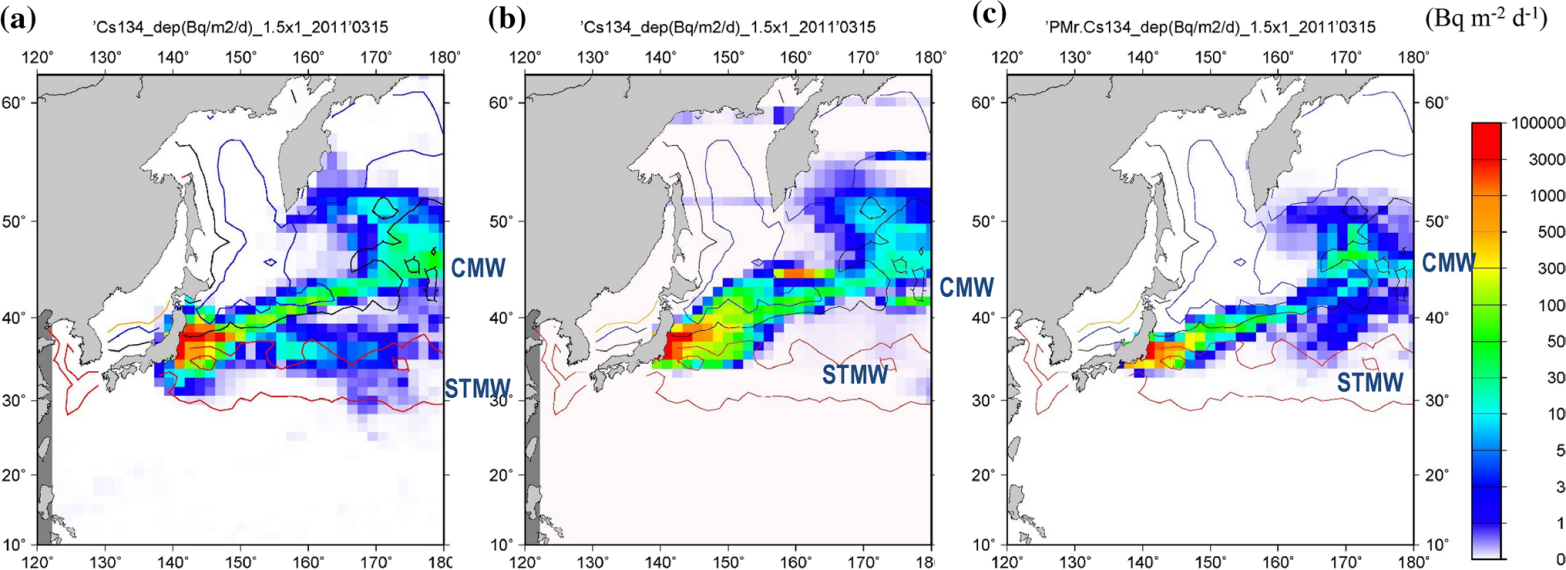
The deposition of ^134^Cs into the North Pacific Ocean on 15 March 2011. **a** The Masingar I model, **b** the Masingar MK-II model, **c** the MRI-PM/r model. The unit of measurement is PBq m^−2^ d^−1^. The data was decay corrected to 11 March 2011. Based on the *σ*_θ_ distributions in the North Pacific Ocean, the deposition region was divided into the STMW and CMW regions

In this study, the estimated OICs134 inventory indicates that approximately 56 ± 10% of the FNPP1-derived radiocaesium was transported eastward in the surface layer in the North Pacific Ocean. The remaining approximate 43% of the FNPP1-derived radiocaesium would be subducted into the ocean interior as the STMW and CMW. Because of very limited data to estimate the subducted amount of radiocaesium in the STMW and CMW, it is difficult to estimate only the measurement data. Then, the horizontal distributions of ^134^Cs deposition in the western North Pacific Ocean were investigated. Figure 2 shows the horizontal distribution of ^134^Cs deposition on 15 March 2011 when the radiocaesium deposition was largest according to the three model simulations. It is noted that the released amount of the FNPP1 ^137^Cs into the atmosphere was similar, although the boundary conditions and physical parameters were different among the models [6]. The feature of the FNPP1-^137^Cs deposition regions and these deposition patterns were almost similar among models, although the detail was different. The largest deposition occurred in the FNPP1-off region, and the higher FNPP1-^134^Cs deposition region extended northeastward in the North Pacific Ocean. The largest deposition region occurred in the STMW and CMW formation regions.

**Fig. 3:**
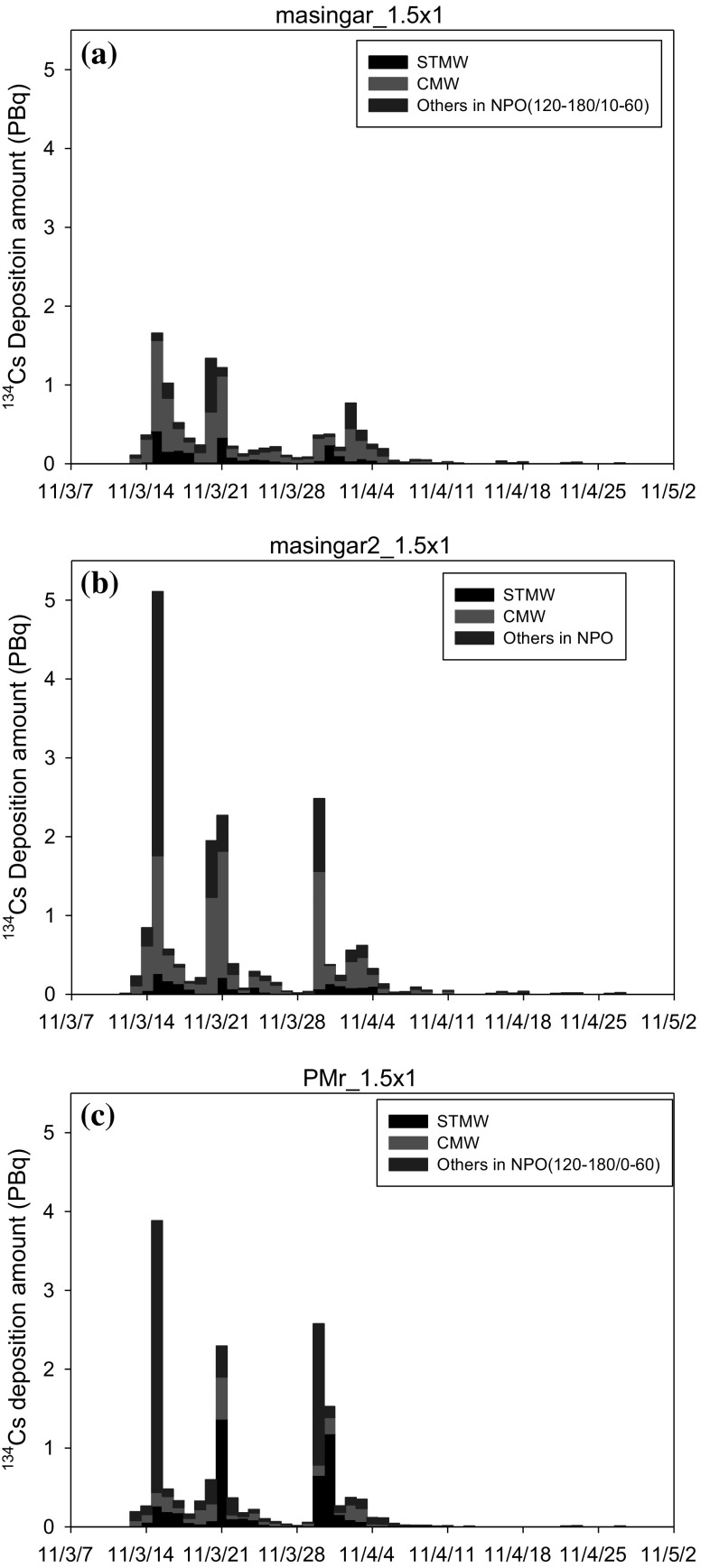
The temporal variation of daily ^134^Cs deposition into the North Pacific Ocean from 11 March to 30 April 2011. The North Pacific Ocean was divided into the STMW, CMW, and other regions. The analytical domain is longitude 120°E–180° between latitude 10°N and 60°N; **a** the Masingar I model, **b** the Masingar MK-II model, **c** the MRI-PM/r model. The data was decay corrected to 11 March 2011. (Color figure online)

The temporal variations of the daily FNPP1-^134^Cs deposition amount in the mode water formation region was also investigated using the three models (Fig. 3). These results are summarized in Table 1. The largest deposition occurred on 15 March, 20–21 March, 29–30 March or 2–3 April 2011. The deposition in the STMW was estimated at 2.2–4.9 PBq, and that in the CMW was estimated at 2.7–10.1 PBq. According to the Masingar model, the FNPP1-^134^Cs deposition was larger in the CMW than that in the STMW.

**Table 1:** The amount of ^134^Cs deposited in the North Pacific Ocean compared with the three models

Region	Classification	Potential density anomaly (*σ*_θ_) (kg m^−3^)	MASINGAR-I (PBq)	MASINGAR MK-II (PBq)	MRI-PM/r (PBq)
NPO	ALL		18.4	18.3	15.2
	Sea		17.16	15.7	12.9
	Land		1.24	2.58	2.28
STMW		25–25.6	3.6	2.2	4.9
CMW		25.6–26.4	9.9	10.1	2.7

The ^134^Cs deposition region in mode water formation region is in the longitude 120°E–180°and latitude 10–60°N based on the JCOPE data

## Discussion

### Evaluation of FNPP1 radiocaesium transported eastward in the surface layer

As described above, the OICs134 amount in the surface layer decay corrected to the accident date was estimated to be 8.6 ± 1.5 PBq. This corresponds to 56 ± 10% of the total amount of released ^134^Cs into the North Pacific Ocean. Although OI results did not cover the whole region in the North Pacific Ocean (Fig. 1), the OI results included the high radiocaesium activity concentration area in the North Pacific Ocean, located in a region from 25°N to 50°N and from 135°E to 135°W in April and May 2011 [1, 5, 8, 9]. Therefore, we think that the underestimation of OICs134 associated with the unavailable OI analysis would be small.

**Fig. 4:**
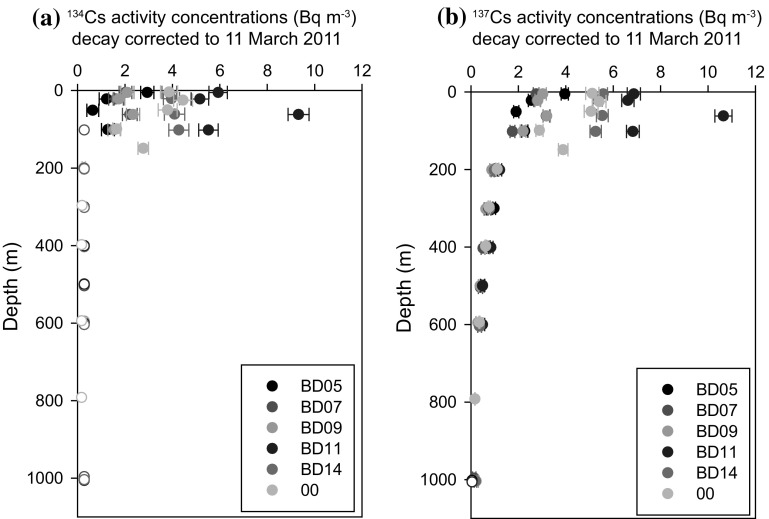
The vertical distributions of (**a**)^134^Cs and (** b**)^137^Cs activity concentrations in the North Pacific Ocean. Data of ^134^Cs and ^137^Cs activity concentrations were decay corrected to 11 March 2011. The sampling stations are BD05 (40.83°N, 149.99°E, 26 August 2012), BD07 (47.00°N, 160.08°E, 26 August 2012), BD09 (47.00°N, 170.58°E, 2 September 2012), BD11 (47.00°N, 180.00°E, 6 September 2012), BD14 (47.00°N, 190.00°E, 11 September 2012), and 00 (47.63°N, 161.83°E, 8 September 2012). Open circles mean that ^134^Cs and ^137^Cs activity concentrations were below the detection limit

**Fig. 5:**
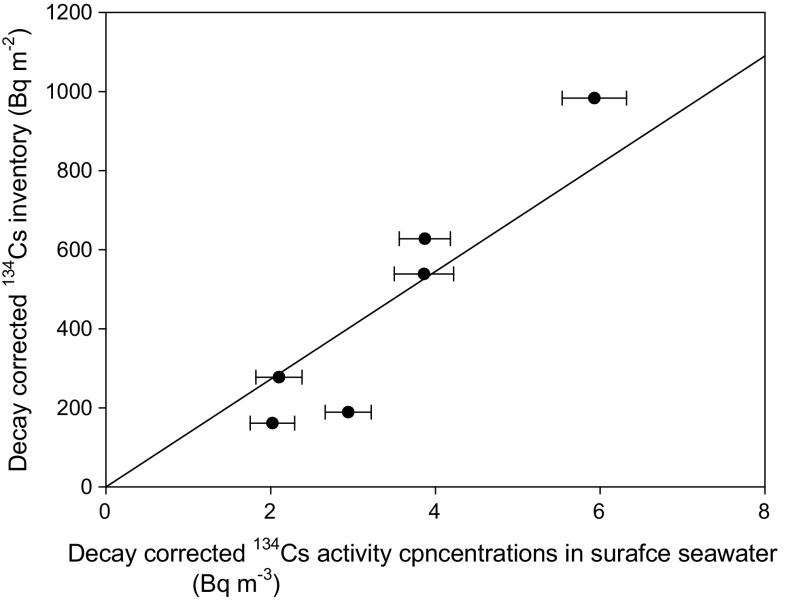
The water column inventory of ^134^Cs decay corrected to 11 March 2011 against the 134Cs activity concentrations in surface seawater

The MPD is also an important factor to estimate the FNPP1-derived radiocaesium amount in the surface layer. In this study, we estimated the MPD based on the vertical profiles of ^134^Cs activity concentrations. Figure 4 shows vertical profiles of ^134^Cs and ^137^Cs activity concentrations in the western North Pacific Ocean in August–September 2012. The activity concentrations of ^134^Cs and ^137^Cs are similar below a depth of 200 m. The ^134^Cs activity concentrations deeper than 200 m were less than the detection limit, suggesting that FNPP1-derived radiocaesium does not penetrate more than a depth of 200 m. For ^137^Cs, it was detected and decreased at depths greater than 200 m. ^137^Cs that measured deeper than 200 m originated from the global fallout. The MPD was estimated by the relationship between ^134^Cs activity concentrations and ^134^Cs inventory as shown in Fig. 5. The MPD was set to a depth of 144 m. Therefore, we consider that our estimation of the amount of radiocaesium in the surface layer would be reasonable.

### Comparison with atmospheric deposition and directly discharged FNPP1 radiocaesium in the mode water formation region

FNPP1-derived radiocaesium released into the North Pacific Ocean originated from atmospheric deposition and direct discharge from the FNPP1. These were transported eastward along the surface currents. The amount of ^134^Cs shown in Table 1 are considered to be only atmospheric deposition. The directly released FNPP1 radiocaesium, which was estimated to be 3.5 ± 0.7 PBq, was mainly transported in the CMW formation region. Added to this directly released amount, the amount of FNPP1-derived radiocaesium in the North Pacific Ocean was 12.4 PBq for the Masingar I model, 13.6 PBq for the Masingar MK-II model, and 6.2 PBq for the MRI-PMr model. Considering that the OICs134 amount in the surface layer was estimated to be 8.6 ± 1.5 PBq, the FNPP1-derived radiocaesium amount transported eastward in the surface layer without subducting into the CMW was approximately 69% and 63% according to the Masingar I and MK-II models, respectively. For the MRI-PMr model, the deposition amount in the mode water formation region was smaller than that transported in the surface layer, which resulted in the deposition of a larger amount out of the JCOPE data region. Furthermore, the deposition into the STMW formation region was larger in the MRI-PMr model than that in the Masingar I and MK-II models. The difference in the deposition amount and deposited region among the three models would reflect the different transport and deposition schemes.

### The fate of FNPP1 radiocaesium subducted into the ocean interior

It was reported that the main body of the FNPP1 radiocaesium-labelled STMW was observed in January 2012, approximately 10 months after the FNPP1 accident, and transported to south of 30°N in November 2012, approximately 20 months after the accident in the North Pacific south of Japan (NPSJ) [21, 49]. As shown in Fig. 3, a larger deposition of FNPP1 radiocaesium occurred three times from March to early April 2011. Considering that seawater was still in the colder season under the strong East Asia winter monsoon from March to early April 2011, the FNPP1 radiocaesium-contaminated surface layer in the STMW formation region, which is south of Kuroshio and the Kuroshio Extension, becomes colder and denser, resulting with the formation of FNPP1 radiocaesium-labelled STMW to be associated with the deep mixed layer. According to the previous research based on the physical parameters in seawater, during the winter, the STMW is capped by the seasonal thermocline and then transported by the southwestward Kuroshio recirculation until approximately 20°N to south and east of Taiwan [50, 51]. It was also found that the FNPP1 radiocaesium-labelled STMW was transported from NPSJ to the East China Sea (ECS), following then the Sea of Japan (SOJ) via the Tsushima Kaikyo, and transported into the North Pacific Ocean via the Tsugaru Strait within several years [52]. The integrated amount of FNPP1-^137^Cs that returned to the North Pacific Ocean through the Tsugaru Strait until 2016 was estimated to be 0.09 ± 0.01 Bq [52], which corresponds to 2.1% of the estimated total amount of FNPP1-^137^Cs in the STMW [23]. This indicates that a small amount of the FNPP1-^134^Cs went back to the surface layer in the North Pacific Ocean.

The subsurface maxima of radiocaesium activity in the CMW was observed in the seawater with *σ*_θ_ of 26.1–26.3 at 34°N–39°N, 165°E from October 2011 to June/July 2012 [1]. These ^134^Cs activity concentrations in the CMW were higher than those in the STMW [1]. This is consistent with this study, in which the FNPP1 radiocaesium amount, which is the sum of the direct release and atmospheric deposition amounts, in the CMW formation region was larger than that in the STMW formation region.

The subducted FNPP1 radiocaesium amount in the ocean interior estimated in this study was approximately 44% of the total amount injected into the North Pacific Ocean, and this estimation is consistent with an estimation from the high resolution ocean modelling study [53]. In Kamidaira’s study, 42.7% of FNPP1-^137^Cs was penetrated below the mixed layer by eddies associated with geostrophic secondary circulations.

## Conclusions

Distributions of the FNPP1 radiocaesium released into the North Pacific Ocean were investigated, and the amounts were estimated in the surface layer, STMW, and CMW in the summer of 2012. The centre of FNPP1-derived contaminated seawater was located in the region approximately 40 and 50°N between 165°E and 178°W. The estimated ^134^Cs in the surface layer was 5.1 ± 0.9 PBq on 1 October 2012 and 8.6 ± 1.5 PBq decay corrected on 11 March 2011. For ^137^Cs, the inventory was estimated to be 13.3 ± 2.3 PBq on 1 October 2012 and 13.9 ± 1.8 PBq on 11 March 2011. Approximately 56 ± 10% of the released amount of ^134^Cs in the North Pacific Ocean was mainly transported eastward in the surface layer. The remaining 44% of the ^134^Cs amount released into the surface seawater was subducted into the ocean interior. Considering that the amount of ^134^Cs injected into the North Pacific Ocean and subducted into the STMW obtained in the previous study were estimated to be 15.3 ± 2.6 PBq and 4.2 ± 1.1 PBq, the amount of ^134^Cs subducted into the CMW was estimated to be 2.5 ± 0.9 PBq based on the mass balance among the three domains; that is, the surface layer, STMW and CMW.

## Electronic supplementary material

Below is the link to the electronic supplementary material.

Supplementary material 1 (PPTX 258 kb)

Supplementary material 2 (XLSX 58 kb)
