# Supplementary material for: Estimate of Fukushima-derived radiocaesium in the North Pacific Ocean in summer 2012
Source: J Radioanal Nucl Chem. 2018 Nov 10;318(3):1587–96. doi: 10.1007/s10967-018-6249-7 (PMC6267120; doi:10.1007/s10967-018-6249-7)
Supplement: Supplementary file 1 — Supplementary material 1 (PPTX 258 kb) [file 10967_2018_6249_MOESM1_ESM.pptx]

## Slide 1
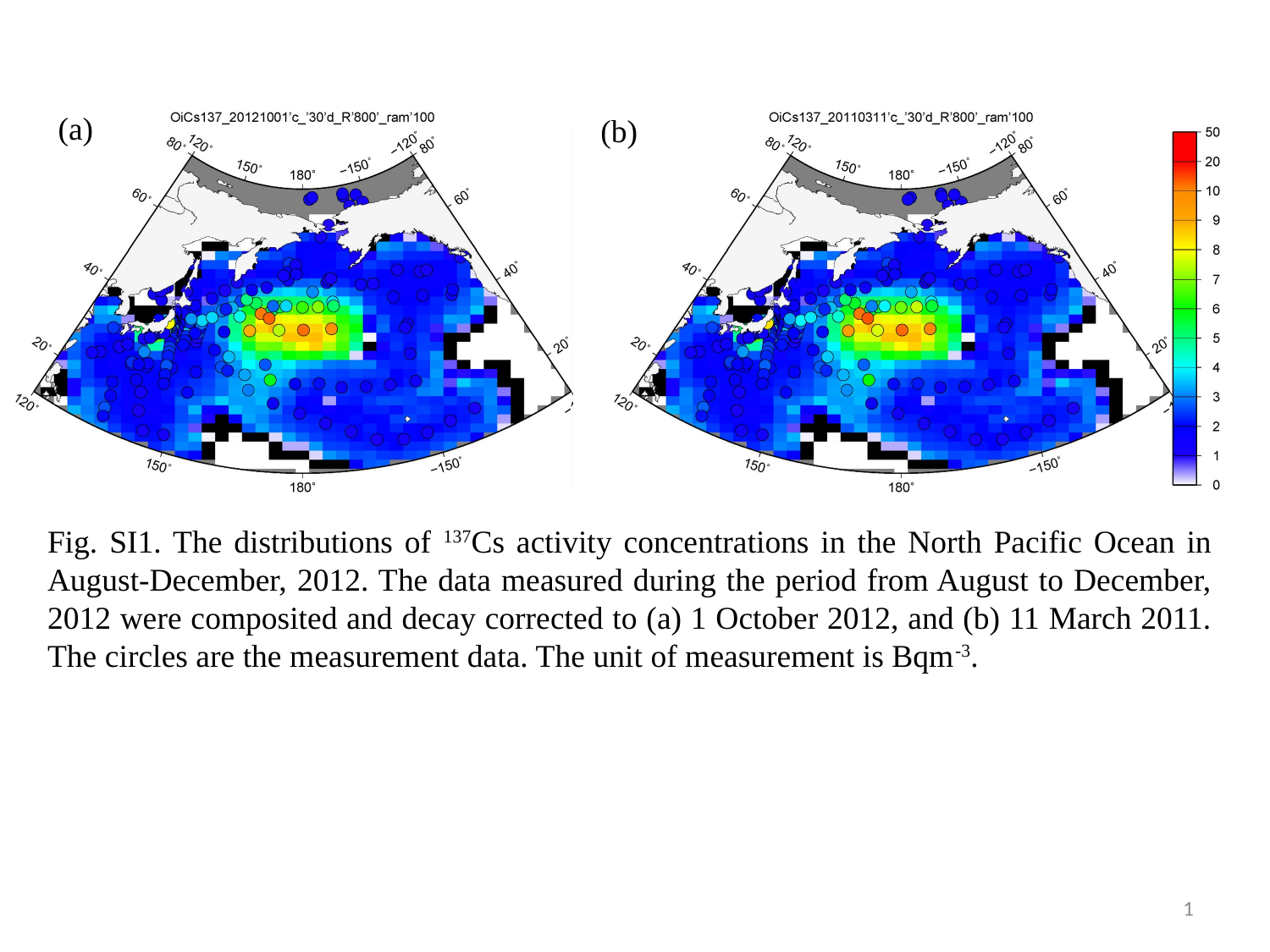

(a)
(b)
Fig. SI1. The distributions of 137Cs activity concentrations in the North Pacific Ocean in August-December, 2012. The data measured during the period from August to December, 2012 were composited and decay corrected to (a) 1 October 2012, and (b) 11 March 2011. The circles are the measurement data. The unit of measurement is Bqm-3.
1
